# Strong Local Scientific Communities Are Essential to Reach the Millennium Development Goals

**DOI:** 10.1371/journal.pntd.0004136

**Published:** 2015-10-29

**Authors:** Serap Aksoy

**Affiliations:** Department of Epidemiology of Microbial Diseases, Yale School of Public Health, Yale University, New Haven, Connecticut, United States of America; University of Washington, UNITED STATES

Infectious diseases disproportionately affect the health of populations living in low- and middle-income countries. In addition to the most prevalent diseases, malaria, tuberculosis (TB), and HIV, more than 1 billion people worldwide—most of whom live in remote, rural areas, urban slums, or conflict zones—are also vulnerable to a diverse group of diseases termed neglected tropical diseases (NTDs). Among the 17 diseases classified as NTDs by the World Health Organization are Chagas disease, human African trypanosomiasis, and leishmaniases caused by protozoa; Buruli ulcer, leprosy, trachoma, and yaws caused by bacteria; cysticercosis, dracunculiasis, echinococcosis, trematodiases, lynpharic filariasis, onchocerciasis, and schistosomiasis caused by helminthes; and dengue, chikungunya, and rabies caused by viruses [1]. Besides their immediate public health concern, NTDs can also be chronic in nature, thriving and promoting poverty among the poorest populations [2]. Hence, there is growing recognition that control of NTDs represents a development opportunity that can help alleviate poverty among the world’s economically challenged peoples. In particular, elimination of NTDs can have direct impact on the achievement of the United Nations Millennium Development Goals, which are targets set in 2000 by the United Nations General Assembly to achieve global economic and health equality to improve people’s lives and end poverty [3]. In addition, one of the three statements issued by the joint Science Academies of Group of Seven (G7) nations in April 2015 also concerned NTDs [4]. The G7 Academies call for:

1. increasing efforts to empower and build capacity in affected countries to deal with these diseases,
2. intensifying research on NTDs,
3. developing and delivering affordable and accessible treatments, and
4. fully accounting for NTDs in the Sustainable Development Goals.

During the last 15 years, improvements in effective public health interventions have led to significant advances, which are reflected in the global malaria incidence rate falling by an estimated 37% and the global malaria mortality rate decreasing by 58% [3]. TB and HIV control efforts have also led to similar major achievements, particularly in sub-Saharan Africa [3]. Continued, scaled-up implementation of these interventions, including diagnostic testing and treatment, will now be necessary to achieve the sustainable disease elimination goals planned for 2020. During the next phase, disease-endemic country (DEC) scientists and local health personnel will undoubtedly play the most significant role. Besides sustained political commitment, predictable financing, and strategic investments in health systems, DEC scientists and health personnel will be critical for conducting effective disease surveillance programs in the affected areas and for implementing strategies and applying new tools that to date have been largely created in the developed country laboratories through various partnerships. Thus, the presence of strong local scientific communities in DECs is essential.

In fact, various initiatives have already been established to foster the development of effective scientific communities in DECs. To encourage and support this process, the 8th African Union Summit held in Addis Ababa in 2007 endorsed the Declaration on Science, Technology, and Scientific Research for Development (Assembly/AU/Decl.5 [VIII]) [5]. Through this declaration the Assembly committed to:

1. encourage more African youth to take up studies in science, technology, and engineering, and invite member states to pay special attention to the teaching of science and technology (S&T);
2. promote and support research and innovation activities, along with the requisite human and institutional capacities; and
3. ensure the enhanced role and revitalization of African universities and other African institutions of higher education, as well as scientific research institutions, so that they can play an effective role as loci of science, technology, engineering education, and development—and also contribute to public understanding of science and technology.

Large-scale initiatives led by key institutions that support scientific work in Africa—including the Wellcome Trust, Royal Society, and Department for International Development (DFID) in the United Kingdom; National Institutes of Health (NIH) and the NIH Fogarty International Center in the United States; as well as private philanthropic institutions such as the Bill and Melinda Gates Foundation and Rockefeller Foundation, among others—have strengthened institutional, academic, and individual scientific capacity, notably in the fields of medicine and agriculture. Collectively, these efforts have helped build considerable scientific infrastructure in DECs and led to the establishment of many successful collaborations and partnerships. It is now essential that both local scientific communities and DEC politicians demonstrate strong ownership of and leadership in the financial, political, and scientific arenas to ensure that the disease elimination programs and targets set for post-2015 can be realized.

One of the essential tenets of a strong scientific community is the ability to participate in the global scientific network as editors and peer reviewers, which are practices that provide scientists the opportunity to exchange ideas and establish global collaborations with their peers. In addition to publishing in international journals, the presence of local journals that practice best principles of publication and peer-review ethics is important for disseminating information, which is often vital for the development of evidence-based local health policies. The global publishing houses should play a major role towards building strong scientific communities in DECs by including scientists and scholars of these communities on their editorial boards, recruiting reviewers from DECs when appropriate, and by providing support for disseminating information on good manuscript writing practices and the publication process. According to the 2015 World Bank report on global S&T indicators, the number of scientific and technical journal publications contributed in 2011 ranged from 413,799 coming from high-income countries to 14,704 from Latin America, 13,542 from South Asia, and 5,416 from sub-Saharan Africa [6]. The fact that all DEC scientists combined apparently contributed fewer than 10% of global journal publications is surely not a reflection of the work that goes on in these communities, but rather, likely results from lack of information on publication processes and inadequate and/or incomplete training in manuscript preparation and scientific writing skills.

**Fig 1:**
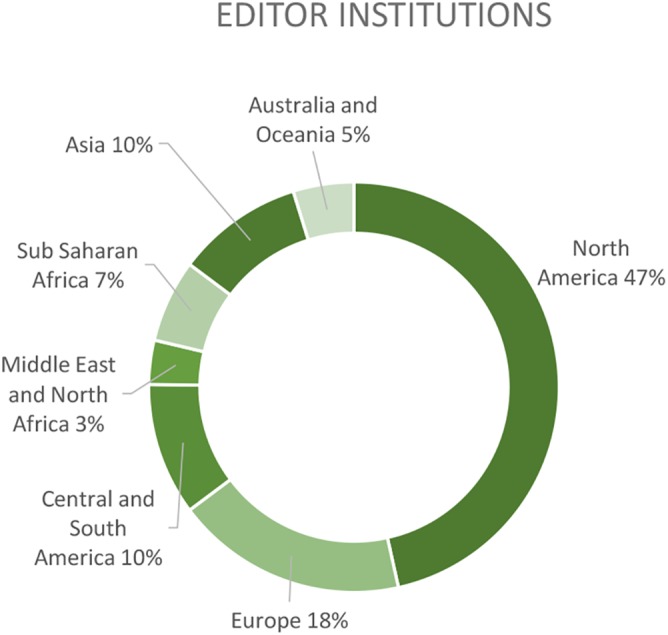
Percent distribution of the PLOS NTDs Editorial Board members according to the different geographic areas they represent.

One of the main goals of *PLOS Neglected Tropical Diseases* is to promote and profile the efforts of scientists in DECs and to help build strong science and health capacity in these countries. We hope to achieve our mission by giving scientists open access to essential information and by providing a venue for publishing their research. Scientists from DECs make up 35% of our Editorial Board (Fig 1). We are also very pleased to report that in 2014, 44% of the authors on the manuscripts published in *PLOS Neglected Tropical Diseases* were from middle- and low-income countries. Of the submitting authors in 2014, 15% were from institutions in Latin America, 13% from East Asia and Pacific, and 10% were from sub-Saharan Africa. Thirty-eight percent of submitting authors in Latin America, 41% of submitting authors in East Asia and Pacific, and 42% of submitting authors in sub-Saharan Africa had their works accepted for publication.

We are keen to see the involvement of junior scientists as well as women in our Editorial Board activities. A highlight of our capacity-building efforts are the “Manuscript Writing Workshops” held by our deputy editors and associate editors in the various international meetings in which they participate, as well as in their overseas work sites. We have a new Resource page in the journal that describes the commitment of our Editorial Board to global capacity-building efforts in publication practices. We included a presentation, which many of us have used in the “Manuscript Writing Workshop,” for other interested editors to download and modify accordingly for their own use [7]. The presentation highlights some of the most common mistakes that result in rejection of manuscripts. It also gives some tips on effective writing skills and the scientific format we follow for research articles. In addition, it provides information—especially aimed at junior scientists—on the various editorial resources available to help improve manuscript writing skills. Besides the workshops, *PLOS Neglected Tropical Diseases* is eager to engage with our peer journals in DECs and share our experience with them to enhance their own platforms. Towards this goal, I have held several Editor Workshops with my peers in local journal editorial boards to share and exchange editorial practices and peer-review processes. To date, the responses we received from the local DEC communities—students and researchers alike—have been very enthusiastic regarding these workshops. On a personal note, each of the workshops I have conducted has been a delightful learning experience for me. I invite more of our esteemed publishing houses, journals, and editorial colleagues to contribute to efforts to reverse the trend on global publication statistics.
